# A probable aculeacin A acylase from the *Ralstonia solanacearum *GMI1000 is *N*-acyl-homoserine lactone acylase with quorum-quenching activity

**DOI:** 10.1186/1471-2180-9-89

**Published:** 2009-05-09

**Authors:** Chin-Nung Chen, Chii-Jaan Chen, Chen-Ting Liao, Chia-Yin Lee

**Affiliations:** 1 Department of Agricultural Chemistry, National Taiwan University, Taipei 10617, Taiwan, R.O.C.

## Abstract

**Background:**

The infection and virulence functions of diverse plant and animal pathogens that possess quorum sensing systems are regulated by *N*-acylhomoserine lactones (AHLs) acting as signal molecules. AHL-acylase is a quorum quenching enzyme and degrades AHLs by removing the fatty acid side chain from the homoserine lactone ring of AHLs. This blocks AHL accumulation and pathogenic phenotypes in quorum sensing bacteria.

**Results:**

An *aac *gene of undemonstrated function from *Ralstonia solanacearum *GMI1000 was cloned, expressed in *Escherichia coli*; it inactivated four AHLs that were tested. The sequence of the 795 amino acid polypeptide was considerably similar to the AHL-acylase from *Ralstonia *sp. XJ12B with 83% identity match and shared 39% identity with an aculeacin A acylase precursor from the gram-positive actinomycete *Actinoplanes utahensis*. Aculeacin A is a neutral lipopeptide antibiotic and an antifungal drug. An electrospray ionisation mass spectrometry (ESI-MS) analysis verified that Aac hydrolysed the amide bond of AHL, releasing homoserine lactone and the corresponding fatty acids. However, ESI-MS analysis demonstrated that the Aac could not catalyze the hydrolysis of the palmitoyl moiety of the aculeacin A. Moreover, the results of MIC test of aculeacin A suggest that Aac could not deacylate aculeacin A. The specificity of Aac for AHLs showed a greater preference for long acyl chains than for short acyl chains. Heterologous expression of the *aac *gene in *Chromobacterium violaceum *CV026 effectively inhibited violacein and chitinase activity, both of which were regulated by the quorum-sensing mechanism. These results indicated that Aac could control AHL-dependent pathogenicity.

**Conclusion:**

This is the first study to find an AHL-acylase in a phytopathogen. Our data provide direct evidence that the functioning of the *aac *gene (NP520668) of *R. solanacearum *GMI1000 is via AHL-acylase and not via aculeacin A acylase. Since Aac is a therapeutic potential quorum-quenching agent, its further biotechnological applications in agriculture, clinical and bio-industrial fields should be evaluated in the near future.

## Background

A bacterial cell-to-cell communication mechanism, quorum sensing, is a regulatory process that utilises small, diffusible signal molecules to modulate specific gene expression in a population density-dependent manner [1,2]. Diverse gram-negative bacteria can synthesise *N*-acyl-homoserine lactones (AHLs) as quorum-sensing signal molecules by means of LuxI-type AHL synthases [3]. These quorum-sensing signal molecules share identical homoserine lactone moieties but vary in length or the carbon substitution on the third position on the acyl side chain. As the population density increases, the AHLs bind to LuxR transcriptional regulators; then, the LuxR/AHL complexes regulate the expression of the target genes. The AHL-mediated quorum sensing mechanisms are highly conserved and could regulate infections and virulence factors in several human and plant pathogenic bacteria, such as *Chromobacterium violaceum*, *Burkholderia cepacia*, *Erwinia carotovora*, *Brucella melitensis*, and *Pseudomonas aeruginosa *[3-5]. Recently, the AHL-mediated quorum-sensing systems have been viewed as new targets for anti-infective therapies. In contrast to traditional drug designs that are either bactericidal or bacteriostatic, the disruption of the AHL-mediated quorum sensing mechanisms, known as quorum quenching, aims to shut down the expression of virulence rather than to kill the organisms. Therefore, quorum quenching has the potential to overcome drug related toxicities, complicating superinfections, and antibiotic resistance in antibiotic therapy [4,6-8].

There are several quorum-quenching strategies available for disrupting the AHL-based quorum-sensing microorganisms, including the enzymatic inactivation of AHL molecules and the inhibition of AHL synthesis by triclosans [9,10]. Another strategy is to block the formation of LuxR/AHL complexes by using halogenated furanones [11]. However, the major quorum-quenching approach for controlling AHL-regulated disease focuses on the AHL-lactonases and AHL-acylases [12]. AHL-acylases degrade AHLs by hydrolysing the amide linkages between the fatty acid chain and the homoserine lactone moiety [13]. To date, only five AHL-acylase genes, i.e. *aiiD *in *Ralstonia *sp XJ12B [14], *ahlM *in *Streptomyces *sp. M664 [13], *pvdQ *and *quiP *in *P. aeruginosa *PAO1 [15-17], and *aiiC *in *Anabaena *sp. PCC7120 [18] have been identified.

Interestingly, the human opportunistic pathogen *P. aeruginosa *PAO1 produces two major AHLs, including *N*-(3-oxo-dodecanoyl)-homoserine lactone (3OC12-HSL) and *N*-butanoyl-homoserine lactone (C4-HSL) [19-21], as well as an AHL-acylase PvdQ; this seemingly different from the common single set of the *luxI*/*luxR *homologue system. *P. aeruginosa *PAO1 possesses a more complex hierarchical AHL mediated quorum-sensing mechanism that is composed of two sets of *luxI*/*luxR *homologues, termed *lasR*/*lasI and rhlR*/*rhlI *systems [19]. These systems are first operated by 3OC12-HSL and C4-HSL, respectively; furthermore, the *lasR/lasI *system can regulate the *rhlR/rhlI *system at the transcriptional and post-translational levels [20,21]. It has been reported that the PvdQ acylase degrades only AHLs with long acyl-chains (3OC12-HSL) and not those with short acyl-chains (C4-HSL) [16]. The co-existence of AHLs with an AHL-degrading enzyme in *P. aeruginosa *PAO1 has been suggested for fine-tuning the expression of virulent genes by manipulating the ratios of their two AHL signals [12].

*Ralstonia solanacearum *is an important soil-borne plant pathogen with an extensive host range. It generally causes severe bacterial wilt disease in many economic crops, including tomato, potato, tobacco, peanut, and banana [22]. *R*. *solanacearum *utilizes a complex hierarchical PhcA regulatory network to control its virulence factors [23]. The PhcA as the central transcriptional regulator in this global regulation network is modulated by 3-OH-palmitic acid methyl ester (3-OH-PAME) [24,25]. *R*. *solanacearum *also possesses a *solI*/*solR *quorum-sensing system that is a *luxI*/*luxR *homologue and is up-regulated by 3-OH-PAME [26]. Inactivation of *solIR *eliminates the synthesis of C6- and C8- HSLs, but does not affect disease or virulence factor production. At least one gene, *aidA *with unknown function, is activated by *solR *[25]. The role of AHLs in *R*. *solanacearum *gene regulation requires much further investigation. However, no report concerning any AHL-degrading enzyme from *R. solanacearum *has been published so far. In this study, an undemonstrated function of the *aac *sequence of *R. solanacearum*GMI1000 homologous to the AiiD acylase was cloned and characterised. The potential of AHL-degrading enzyme is also discussed here.

## Methods

### Bacterial strains, culture media, and conditions

All bacterial strains and plasmids used in this study are listed in Table 1. The bioassay strain of *C. violaceum *CV026 [27] used is mini-Tn5 mutant derived from the wild type strain *C. violaceum *ATCC 31532 and defective in C6-HSL production. *E*. *coli *DH10B (Invitrogen Ltd, California, USA) was used as a blue-white screening host. *E. coli *BL21(DE3) (Novagen Ltd, Wisconsin, USA) was used as a host for large scale protein expression. *E. coli *CA027ZC09 that harbours pZC09 as the *R. solanacearum*GMI1000 *aac *gene donor was used to perform gene cloning [28]. *C. violaceum *and *E. coli *were cultured in Luria Bertani (LB) broth or LB agar plates at 30°C and 37°C, respectively. *Candida tropicalis *F-129 [29] was cultured in LB broth at 37°C for minimum inhibitory concentration (MIC) tests. When required, antibiotics were incorporated into the growth medium in the following concentrations: ampicillin (100 μg·ml^-1^), tetracycline (10 μg·ml^-1^), kanamycin (50 μg·ml^-1^), and streptomycin (10 μg·ml^-1^).

**Table 1:** Bacterial strains and plasmids used in this study

Strain or plasmid	Genotype or Description^a^	Reference
Strains		
*C. violaceum *CV026	White indicator strain; *cviI*::Tn5 *xylE*; Amp^r^, Kan^r^, Str^r^, Tet^s^, Ery^s^, Chl^s^	27
*E. coli *CA027ZC09	The genomic clone generated from *Ralstonia solanacearum *GMI1000 for sequencing harbor plasmid pZC09 containing *aac *gene (RSc2547); Amp^r^	INRA-CNRS^b^
*E. coli *DH10B	*F*^-^*mcrAΔ*(*mrr*-*hsdRMS*-*mcrBC*) *Φ80lacZΔM15 ΔlacX74 deoR recA*1 *endA1 araΔ139 Δ*(*ara leu*)*7697 galU galK λ*^-^*rpsL nupG*; Str^r^	Invitrogen
*E. coli *BL21(DE3)	*hsdS gal *(*λ*cI*ts*857 *ind1 Sam7 nin*5 *lacUV5*-T7 gene 1)	Novagen
*Candida tropicalis *F-129	Test strain for the MIC of aculeacin A assay	29
Plasmids		
pZC09	Plasmid generated from *Ralstonia solanacearum *GMI1000 for sequencing project from which the *aac *gene was amplified; Amp^r^	INRA-CNRS^b^
pBBR1MCS-3	Mobilisable broad-host-range cloning vector; low copy number; *mol*, *rep*, *lacZ; *Tet^r^	30
pS3aac	Transcriptional fusion of *aac *gene in pBBR1MCS-3; Tet^r^	This study
pET21a	Expression vector; T7 promoter; C-terminal HisTag; *lacI*; Amp^r^	Novagen
pET21aac	Translational fusion of *aac *gene in pET21a; Amp^r^	This study

^a ^Amp: ampicillin; Kan: kanamycin; Tet: tetracycline; Nal: nalidixic acid; Str:

streptomycin; Chl: chloramphenicol; Ery: erythomycin

^b ^INRA-CNRS: Laboratoire de Biologie Moleculaire des Relations Plantes

Microorganismes INRA-CNRS, France

### *In vitro *whole cell bioassay for AHL-degrading activity

The bioassay was modified from the method used for the isolation of AHL-degrading *Streptomyces *strains [13]. For preparation of the well plate [27], 10 ml of LB agar containing 1.5% agar was seeded with 1 ml of *C. violaceum *CV026 overnight culture, and then immediately poured over the surface of solidified LB agar. After the overlaid agar solidified, several wells were punched on the top of the LB agar to form the well plate. For preparation of the whole cell reaction mixture, 1 ml of *E. coli *clone overnight culture was centrifuged and suspended in 1 ml of 100 mM Tris buffer (pH 7.0). Then, 150 μl of the cell suspension (OD_600 _= 1.2) was mixed with an equal volume of 25 μM *N*-(heptanoyl)-L-homoserine lactone (C7-HSL) or C8-HSL (Fluka Ltd, SG, Switzerland) and incubated at 30°C, with gentle agitation, for 1 h. The whole cell reaction mixture was boiled (95°C, 5 min) to stop the enzymatic reaction. One hundred microlitres of the reaction mixture was loaded into the well on the plate. The loaded bioassay plate was finally incubated in the upright position at 30°C for 24 h to observe whether adequate colour development was achieved. A violet pigmentation of the bacterial lawn distributed around the wells indicated an absence of AHL-degrading activity.

### Cloning and expression of *aac *gene

The plasmid DNA pZC09, carrying the *aac *gene, was prepared by using Gene-Spin Miniprep Purification Kit (Protech Ltd, Taiwan) and used as a PCR template. The *aac *gene was amplified by PCR with primers, 5'-GAGGTACCG**AAGGAG**GACACCGC*ATG*-3' (forward) and 5'-CGACTAGT*TCA*CTGCGACAGCTTTGTCACCT-3' (the *Kpn*I and *Spe*I sites are underlined, the start and stop codons are in italic, the RBS site is in bold font). Template DNA (10 ng) was added to the PCR reactions at a final reaction volume of 50 μl (1× DyNAzyme II buffer, 200 μM deoxynucleotide triphosphate, 1.0 μM primer, 2% dimethyl sulfoxide (Sigma Ltd, MO, USA), and 5.0 U DyNAzyme™ II DNA polymerase (Finnzymes Ltd, ESPOO, Finland). PCR was performed in a GeneAmp PCR system 9700 (Perkin Elmer Ltd, CA, USA). The PCR products were digested with *Kpn*I and *Spe*I and then purified by a PCR-M™ Clean Up System kit (Viogene Ltd, Taiwan). Eighty ng of the purified PCR product was added into 15 μl of the ligation mixture (50 ng of *Kpn*I/*Spe*I-digested pBBR1MCS-3, 1× ligation buffer, and 5 U T4 DNA ligase) and incubated at 16°C for 16 h. The resulting construct, pS3aac, was transformed into *E. coli *DH10B by the heat shock method [31] and screened on LB agar containing tetracycline (10 μg·ml^-1^), isopropyl-β-D-thiogalactopyranoside (IPTG, 50 μg·ml^-1^), and 5-bromo-4-chloro-3-indolyl-D-galactoside (X-Gal, 50 μg·ml^-1^). Then, the positive clones of *E. coli *DH10B (pS3aac) expressing AHL-degrading activity were identified through the *in vitro *whole cell bioassay. Next, the cloned *aac *gene was sequenced by an ABI PRISM 3730XL DNA Analyzer along with an ABI PRISM BigDye Terminator Cycle Sequencing Ready Reaction Kit (Perkin-Elmer).

### Preparation of crude Aac proteins

The *aac *gene was amplified by PCR with the primers 5'-CGCAGAATTCATGACGCACGGATTC-3' (the *Eco*RI site is underlined) and 5'-CGGCAAGCTTCTGCGACAGCTTTG-3' (the *Hin*dIII site is underlined), and then ligated to vector pET21a (Novagen). The resultant pET21aac was transformed into the expression host *E. coli *BL21(DE3). One ml of cultured *E. coli *BL21 (pET21aac) (OD_600 _= 0.6) were induced by using 1.0 mM IPTG for 20 h at 20°C. The harvested cells were resuspended in 0.5 ml of 50 mM sodium phosphate (pH 7.0) and then broken by ultrasonification for 1 min (pulse on, 0.8 s; pulse off, 0.2 s) with a Sonicator^® ^(Heat System, Taiwan). The total proteins were analysed by 6% sodium dodecyl sulphate polyacrylamide gel electrophoresis (SDS-PAGE).

### ESI-MS analysis

To analyse the degradation products of C7-HSL that were digested by *E. coli *(pS3aac), electrospray ionization mass spectrometry (ESI-MS) was performed on a Q-Tof Ultima™ API equipped with a nano-spray Z-spray source (Micromass, UK). One ml of *E. coli *(pS3aac) cells (OD_600 _= 1.2) was washed three times and suspended in 1 ml of 100 mM sodium phosphate buffer (pH 7.0) containing either 0.5 mM C7-HSL or 10 mM ammonia acetate buffer (pH 7.0) containing 0.5 mM C7-HSL, and then each sample was incubated at 30°C for 1 h. The reaction mixtures were centrifuged at 13,000 rpm for 1 min and then the supernatants were collected as the analytic samples. The analytic sample with the sodium phosphate buffer was diluted 100-fold with 0.018% triethylamine (pH 7.0) containing 40% acetonitrile and 10% methanol and was then ionised by positive-ion electrospray (ESI^+^-MS) to detect HSL. The analytic sample with the ammonia acetate buffer was diluted 10-fold with 50% methanol and then ionised by negative-ion electrospray (ESI^-^-MS) to detect heptanoic acid. In order to analyse the degradation products of aculeacin A, i.e. palmatic acid, 40 μl of Aac-digested mixture (6 μg of aculeacin A and 7.2 μg of purified Aac in 10 mM ammonia acetate) was diluted with 40 μl of 50% acetonitrile containing 0.1% formic acid and then detected by ESI^+^-MS.

In this study, we used the following condition for ESI-MS. Approximately 400 nl/min analyte flow rate was used with the Q-Tof instrument. The cone and capillary voltage was set to 135 V and 3.5 KV, respectively, and the source block and desolvation temperature was 80°C and 150°C, respectively. The range of *m/z *value was set to 50 ~500 since this was sufficient for all of degraded products. Data was analyzed by MassLynx 4.0 software (Micromass, UK).

### HSL-OPA assay for AHL-acylase activity

A modified homoserine lactone-*o*-phthaldialdehyde (HSL-OPA) assay was used to quantify the AHL-acylase activity [13]. Seven AHLs (Fluka Ltd, SG, Switzerland) were used as substrates of AHL-acylase. Various AHL-degrading products were collected using the preparation method of the analytic sample in the sodium phosphate buffer, as described in ESI-MS analysis. One hundred μl of each AHL-degrading product was immediately mixed with 100 μl of *o*-phthaldialdehyde reagent solution (OPA, Sigma), and then the mixture was incubated for 2 min at 25°C to prepare the fluorescent derivative of the released HSL. The absorbance of OPA-derivatives was measured at OD_340 _using a U-2000 spectrophotometer (Hitachi Ltd, Tokyo, Japan). A standard HSL with a range of 0.1 ~1 mM was used to calibrate the assay and render a linear correlation: OD_340 _= 0.0014 [HSL] (*r*^2 ^= 0.99). One unit of the AHL-acylase activity is defined as the released nmol amount of HSL after an AHL is digested by 1 ml of cell suspension (OD_600 _= 1.2, cell density reaches 3 × 10^7 ^CFU ml^-1^) at 30°C for 1 min.

### Violacein quantitative assay

To observe the *in vivo *expression of the *aac *gene in *C. violaceum*, the pS3aac was transformed to *C. violaceum *CV026 by the heat shock method [31] and a violacein quantitative assay [32] was performed. One ml of cultured *C*. *violaceum *CV026 (pS3aac) (OD_600 _= 0.7) was added into 100 ml of fresh LB broth containing tetracycline and 0.5 mM C7-HSL, and then incubated at 30°C at 250 rpm for 24 h. At intervals of 2 h, the violacein from 0.5 ml of various interval cells was extracted with 1 ml of 95% ethanol for 1 min. The supernatant containing the violacein was collected by centrifuging at 13,000 rpm for 1 min. The absorbance of the supernatant was measured at a wavelength of 576 nm (OD_576_) using a U-2000 spectrophotometer (Hitachi).

### Chitinase activity assay

The chitinolytic activity assay was modified from the method for detecting chitinolytic activity on agar plates [33]. Cells were seeded on LB agar containing tetracycline (10 μg·ml^-1^), 0.5 mM C7-HSL, and 0.2% (w/v) chitin from crab shells (Sigma). The plate was incubated at 30°C for 3 ~5 d to observe whether a clear zone formed around the colonies. The formation of a clear zone indicated a positive reaction.

### Minimal inhibitory concentration (MIC) of aculeacin A

The assay for the determination of MIC values of aculeacin A was modified from the dilution susceptibility test [34]. A series of samples of 10 ml LB broth containing either aculeacin A or Aac-treated aculeacin A with concentrations in the range of 0–1 μg·ml^-1 ^was prepared and inoculated with 100 μl of 16 h pre-cultured *Candida tropicalis *F-129 and incubated at 37°C for 16 h. The growth of the cells was measured at OD_600_. Serial dilutions of aculeacin A were incubated with 12 μg of purified Aac in 90 μlof sodium phosphate (pH 7.0) at 30°C for 1.5 h; subsequently, the dilution susceptibility test was performed.

### Bioinformatics

The first cloned AHL-lactonase gene *aiiA *[35] and the AHL-acylase gene *aiiD *[14] were utilised as the target genes in the BLASTN and BLASTP programs [36,37] at NCBI. Several public *R. solanacearum*GMI1000 genomic clones containing the *aac *gene were searched by the GMI1000 clone finder. http://bioinfo.genopole-toulouse.prd.fr/annotation/iANT/bacteria/ralsto/index.html.

### Statistics

The Microsoft Excel 2003 *t*-test program was used.

## Results

### Identification of candidate AHL-degrading enzymes encoded by *R. solanacearum*GMI1000

BLASTN and BLASTP searches of the annotated *R. solanacearum*GMI1000 genome sequence (NC 003295) and megaplasmid pGMI1000MP (NC003296) [28] identified a single 2,388-bp *aac *gene (Locus tag RSc2547) with an 83% identity match when interrogated with the AHL-acylase *aiiD *sequence [14]. No significant hits were obtained with the AHL lactonase *aiiA *sequence [35]. However, the *aac *gene encodes a putative protein that was defined as a probable aculeacin A acylase transmembrane protein (NP 520668). Among the function-demonstrating proteins, Aac shared 83%, 39%, 24%, and 24% identities at the peptide level with the AHL-acylase from *Ralstonia *sp. XJ12B [14], aculeacin A acylase from *Actinoplanes utahensis *[38], cephalosporin acylase from *Brevundimonas diminuta *[39], and Penicillin G acylase from *Providencia rettgeri *[40], respectively.

### Cloning and expression of the aac gene of *R. solanacearum*GMI1000

**Figure 1:**
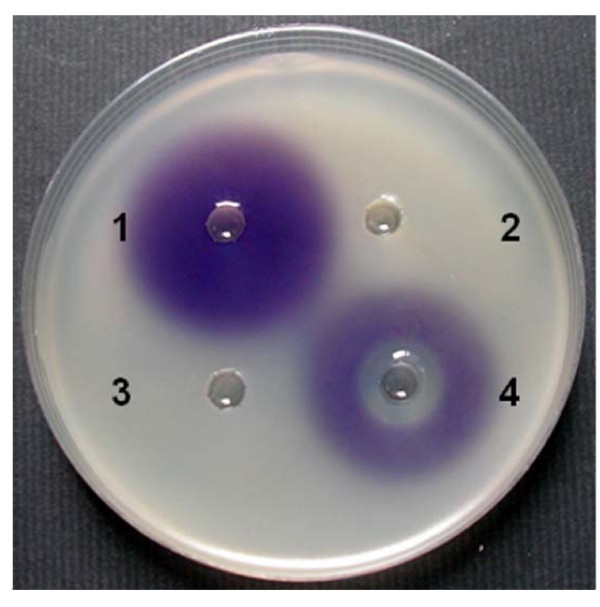
**The Aac acylase degrades C7-HSL in *C. violaceum *CV026 cultures and inhibits violacein production**. The *E. coli *DH10B (pS3aac) overnight culture was centrifuged, and the harvested cells were suspended into 100 mM Tris buffer (pH 7.0). The cell suspensions and cell free supernatants were mixed with 25 μM C7-HSL each and then incubated at 30°C for 1 h. The mixtures were assayed by the *in vitro *whole cell bioassay. Well 1, C7-HSL (AHL-non-degrading control); well 2, Tris buffer (AHL-degrading control); well 3, the mixture of cell suspensions with C7-HSL; well 4, the mixture of supernatants with C7-HSL.

**Figure 2:**
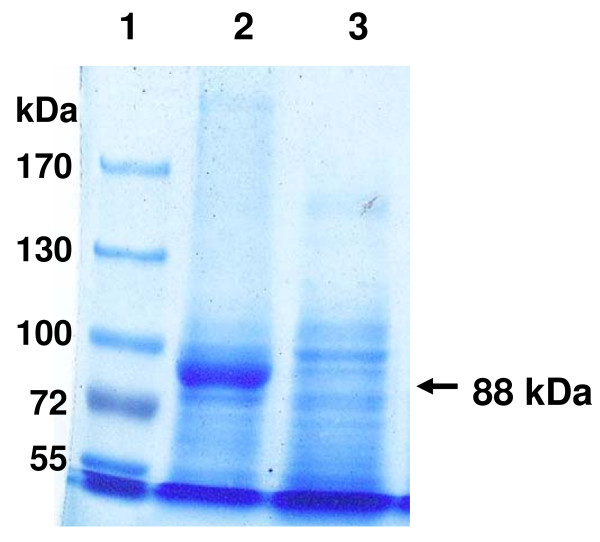
**SDS-PAGE analysis Aac expressed by *E. coli *BL21(DE3)**. The crude proteins were prepared from the recombinant *E. coli *BL21 (pET21aac) and analysed by 6% SDS-PAGE. The arrow indicates the Aac. Lane 1, pre-stained protein ladder marker; lane 2, IPTG-induced crude proteins; lane 3, IPTG-non-induced crude proteins.

The *aac *gene was PCR amplified (refer to Materials and Methods) and the 2,405 bp product was cloned in pBBR1MCS-3 to yield plasmid pS3aac. To analyse the ability of Aac to degrade AHLs pS3aac was used to transform *E. coli *DH10B. The cloned *aac *sequence was confirmed to have no mutations. For examining the degrading activity of the clone *E. coli *DH10B (pS3aac), C6-, C7-, and C8- HSLs were used as autoinducers in performing a whole cell bioassay described in the Materials and Methods. The results of the whole cell bioassay revealed that *E. coli *DH10B (pS3aac) cells were inactive against C6-HSL while active against C7- and C8-HSLs (Table 2). Since the vector pBBR1MCS-3 does not contain *lacI*, we considered that *E. coli *DH10B (pS3aac) cells exhibit C7- and C8-HSLs degrading activities inrespective of the presence or absence of IPTG induction (Table 2). Because C7-HSL was a more sensitive AHL than C8-HSL (data not shown), we chose C7-HSL for inducing *C. violaceum *CV026 to produce violacein in whole cell bioassay (Fig. 1, well 1). The cells of *E. coli *DH10B (pS3aac) exhibited C7-HSL degrading activity (Fig. 1, well 3), while no activity was observed in the cell-free culture supernatant of *E. coli *DH10B (pS3aac) (Fig. 1, well 4). This data indicated that the protein encoded by the *aac *gene is a cell associated AHL-degrading enzyme. The *aac *gene was fused into pET21a to yield plasmid pET21aac, and then over expressed in *E.coli *BL21(DE3) from an inducible promoter. The SDS-PAGE analysis demonstrated that the IPTG-induced total proteins contained a polypeptide with a molecular mass of 88 kDa that was consistent with the 824 residues Aac-fused protein that had a predicted molecular mass of 88,645 Da (Fig. 2).

**Table 2:** The AHL-degrading abilities of *E. coli *DH10B (pS3aac) evaluated by whole cell bioassay

	AHL-degrading abilities^a^											
	
	C6-HSL				C7-HSL				C8-HSL			
			
Test strains	IPTG(+)		IPTG(-)		IPTG(+)		IPTG(-)		IPTG(+)		IPTG(-)	
						
	C	S	C	S	C	S	C	S	C	S	C	S
*E. coli *DH10B (pBBR1MCS-3)	**-**	**-**	**-**	**-**	**-**	**-**	**-**	**-**	**-**	**-**	**-**	**-**
*E. coli *DH10B (pS3aac)	**-**	**-**	**-**	**-**	+	**-**	+	**-**	+	**-**	+	**-**

^a ^The whole cells (C) and cell-free supernatants (S) of test strains, were prepared from the 0.4 mM IPTG induced and uninduced cultures, and then reacted with various AHLs to perform whole cell bioassays. Identical to the result in Fig. 1, the absence of a violet lawn indicates a positive AHL-degrading ability and is defined as'+'; a violet lawn indicates no AHL-degrading ability and is defined as'**-**'

### Aac is an AHL-acylase and not an aculeacin A acylase

**Figure 3:**
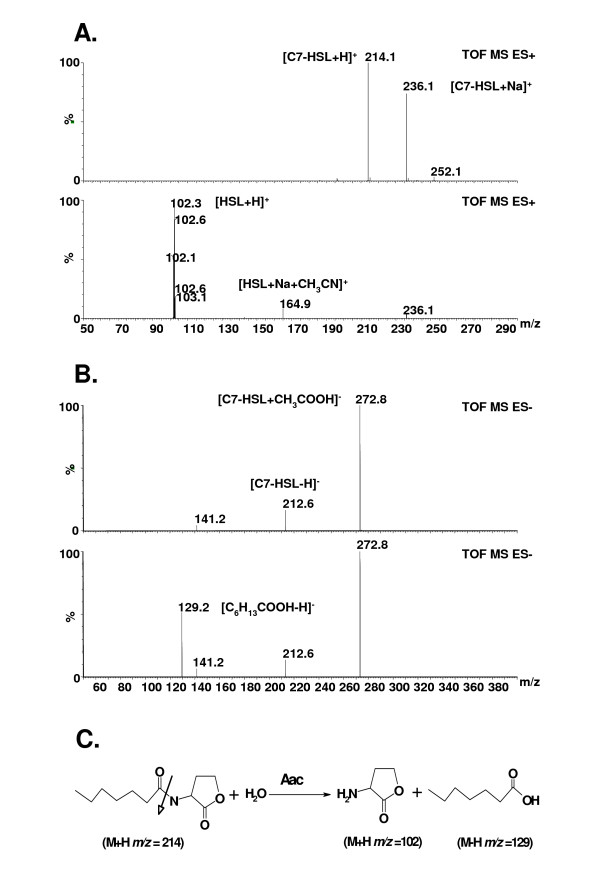
**ESI-MS spectrometry analysis of C7-HSL degradation by AHL-acylase Aac**. The *E. coli *DH10B (pS3aac) cells were suspended in 0.1 mM sodium phosphate and 0.01 mM ammonia acetate, respectively, and then mixed with 25 μM C7-HSL for the degradation reaction described in Materials and Methods. (a) To detect HSL, the ESI^+^-MS spectra of undigested C7-HSL (top) and degraded C7-HSL products (bottom) were shown. (b) To detect heptanoic acid, the ESI^-^-MS spectra of undigested C7-HSL (top) and degraded C7-HSL products (bottom) were shown. (c) Mechanism of C7-AHL degradation by Aac. The white arrow indicates the Aac catalytic site.

To demonstrate whether the Aac protein is an AHL-acylase, we performed the ESI-MS analysis. *E. coli *DH10B (pS3aac) cells were first reacted with C7-HSL at 30°C for 60 min. If the enzyme encoded by the *aac *gene is an AHL-acylase, we predicted that two free digested products, HSL and heptanoic acid, would be detected. Since ESI^+^-MS could not detect the carboxylic group (COO^-^), only HSL was detectable. The fatty acids containing the carboxylic group would have to be detected by ESI^-^-MS. The analytic results showed that C7-HSL (M+H *m/z *= 214) could be digested into HSL (M+H *m/z *= 102) and heptanoic acid (M-H *m/z *= 129) (Fig. 3). We also observed that the amount of the heptanoic acid gradually increased, starting from the 15^th ^min until the 60^th ^min of reaction times (data not shown). Thus, our results indicate that the *aac *gene encodes an AHL-acylase.

Since Aac was predicted as a probable aculeacin A acylase transmembrane protein, we compared the MICs of aculeacin A and Aac-treated aculeacin A in order to determine whether the Aac protein functioned similar to aculeacin A acylase. As shown in Table 3, the MICs of both aculeacin A and Aac-treated aculeacin A for *Candida tropicalis *F-129 were 0.05 μg·ml^-1^. In addition, the predicted Aac-digested products of aculeacin A, i.e. palmatic acid (M+H *m/z *= 257), were not found in the ESI-MS analysis (data not shown). These results revealed that Aac is not an aculeacin A acylase.

**Table 3:** The minimal inhibitory concentrations of aculeacin A for *Candida tropicalis *F-129

	OD_600_^a ^at serial diluted aculeacin A (μg·ml^-1^)							
	
Aculeacin A	0	0.001	0.005	0.01	0.05	0.1	0.5	1
Untreated	0.592 ± 0.036*	0.615 ± 0.088*	0.255 ± 0.096*	0.126 ± 0.029*	0.045 ± 0.006	0.047 ± 0.008	0.043 ± 0.002	0.041 ± 0.004
Aac treated	0.629 ± 0.032*	0.634 ± 0.047*	0.297 ± 0.030*	0.093 ± 0.017*	0.070 ± 0.035	0.054 ± 0.007	0.044 ± 0.002	0.042 ± 0.005

^a ^Values represent the averages ± standard errors from triplicate independent assays. By one-tailed student's *t*-test (*P *< 0.05), the value with an asterisk (*) designates a significant difference from the 1 μg·ml^-1 ^of aculeacin A test shown in the same row. There is no significant difference between Aac-untreated aculeacin A and Aac-treated aculeacin A in the same column

### Aac is active against AHLs with acyl side chains greater than 6 carbons

To determine the substrate specificity and enzymatic activity of the AHL-acylase Aac in *E. coli *DH10B (pS3aac), a range of AHLs were mixed with cells of *E. coli *DH10B (pS3aac) to perform the HSL-OPA assay. As shown in Table 4, *E. coli *DH10B (pS3aac) could not degrade the short-chain AHLs, C4-, C6-, or 3OC6-HSLs; however, *E. coli *DH10B (pS3aac) exhibited activities against long-chain AHLs, C7-, C8-, 3OC8-, C10-HSLs. The AHLs of more than ten carbon-acyl chains, i.e. C12-HSL and C14-HSL, could not be determined due to the poor solubility of their substrate. These results indicate that the substrate specificity of the AHL-acylase Aac is within the limit of more than six carbon-acyl chain AHLs.

**Table 4:** Substrate specificities of the AHL-acylase Aac against AHLs

	AHL-acylase activities (nmol·min^-1^·ml^-1^)^a^	
	
AHLs	*E. coli *DH10B (pBBR1MCS-3)	*E. coli *DH10B (pS3aac)
C4-HSL	0.26 ± 0.15 (7.2%)	0.37 ± 0.13 (10.3%)
C6-HSL	0.31 ± 0.15 (8.7%)	0.38 ± 0.10 (10.5%)
3OC6-HSL	0.37 ± 0.09 (10.5%)	0.35 ± 0.09 (10.5%)
C7-HSL	0.23 ± 0.15 (6.4%)	3.60 ± 0.31 (100.0%)*
C8-HSL	0.21 ± 0.16 (5.7%)	1.63 ± 0.21 (45.4%)*
3OC8-HSL	0.22 ± 0.17 (6.1%)	2.56 ± 0.04 (71.1%)*
C10-HSL	0.25 ± 0.15 (7.1%)	3.10 ± 0.25 (86.1%)*

^a ^Values represent the averages ± standard errors from triplicate independent HSL-OPA assays. The relative activity in parentheses is based on defining C7-HSL-degrading activity exhibited by *E. coli *DH10B (pS3aac) as 100%. The value with an asterisk (*) is the significant difference from the *E. coli *DH10B (pBBR1MCS-3) shown in the same row by one-tailed Student's *t*-test (*P *< 0.05)

### Expression of the *aac *gene in *C. violaceum *CV026 inhibited the production of violacein and chitinase activity that were observed in the AHL-mediated phenotype

**Figure 4:**
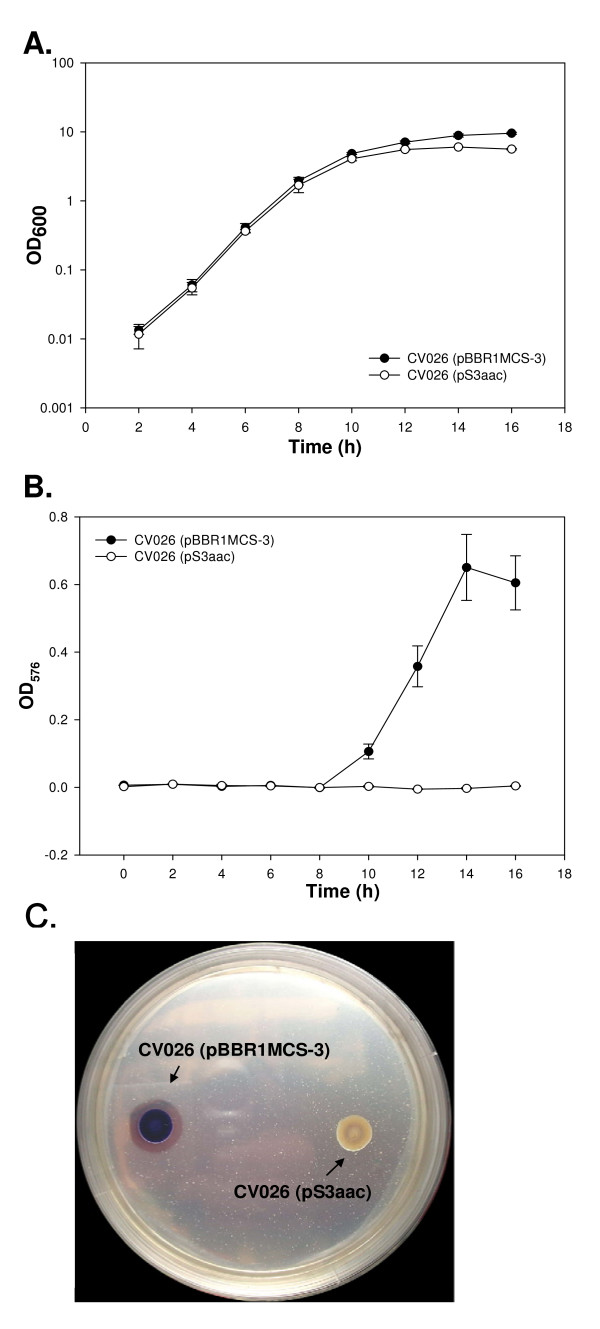
**The effects of Aac on the production of violacein and chitinase activity in *C. violaceum *CV026**. The plasmids pBBR1MCS-3 and pS3aac were transformed into *C. violaceum *CV026. Both of them were cultivated in LB containing tetracycline as well as 25 μM C7-HSL. (a) Cell growth was monitored by measuring the OD_600_. (b) The violacein production was determined by OD_576 _during growth. The data represent the mean values of three independent experiments. (c) The overnight cultures of *C. violaceum *CV026 (pS3aac) and *C. violaceum *CV026 (pBBR1MCS-3) (no *aac *insert) were seeded onto an LA plate containing tetracycline, C7-HSL and chitin in order to assay the chitinolytic activity. The plates were incubated at 30°C for 5 d. The formation of a clear zone around the colonies indicated positive chitinolytic activity.

To examine whether the *aac *gene has the potential of being a quorum-quenching agent, an AHL regulated strain, *C. violaceum *CV026, was used as a target microorganism. The mutant *C. violaceum *CV026 cannot produce violacein unless provided with exogenous AHL [27]. Therefore the pS3aac was transformed into *C. violaceum *CV026 to observe whether violacein production was reduced during culture with exogenous AHL. As shown in Fig. 4A, the result indicates that the expression of the *aac *gene did not influence the growth of *C. violaceum *CV026 during the late exponential phase but slightly influenced its growth during the stationary phase. Interestingly, *C. violaceum *CV026 (pBBR1MCS-3) produced violacein after the late exponential phase, while *C. violaceum *CV026 (pS3aac) completely failed in producing violacein (Fig. 4B). Since it was reported that chitinases could be regulated by endogenous C6-HSL in *C. violaceum *ATCC 31532 [33], we decided to evaluate the chitinolytic activity of *C. violaceum *CV026 (pS3aac). *C. violaceum *CV026 (pBBR1MCS-3) was able to form clear zones on LB agar containing tetracycline, chitin, and C7-HSL. However, no clear zone were observed around the *C. violaceum *CV026 (pS3aac) colonies (Fig. 4C). These results indicated that transferring the *aac *gene into *C. violaceum *CV026 significantly inhibited violacein production and chitinase activity.

## Discussion

We successfully subcloned and identified an *aac *gene (NP 520668) from *R. solanacearum*GMI1000 as an AHL-acylase that did not degrade aculeacin A, ampicillin, and ceftazidime (data not shown). The amino acid sequence of Aac is similar to that of AHL-acylase from *Ralstonia *sp. XJ12B (*Ralstonia eutropha*) with 83% identity. However, this is the first study to report the presence of an AHL-acylase in a phytopathogen.

To verify the existence of an AHL-acylase, both gas chromatography assays [16] and HPLC-ESI-MS analyses [13,14] are generally used to analyse the digested AHL products. Our report provides a simple and rapid ESI-MS analysis to verify AHL-acylase. To identify an AHL-acylase, two target products, the released HSL and the corresponding fatty acid, were detected when the AHL was digested by the AHL-degrading clone. Since the released fatty acids would be further metabolized by β-oxidation during cultivation [41], excessively long digestion times should be avoided. The digestion mixture was directly used as a sample to perform ESI-MS analysis. The reaction buffers were observed to have a decisive effect on ESI-MS analysis. When 100 mM sodium phosphate (pH 7.0) was used as a reaction buffer, only the phosphate ([M-H] m/z = 97) was found in the ESI^-^-MS pattern, wherein the fatty acid was still not detectable (data not shown). In contrast, when 10 mM ammonia acetate was used as a reaction buffer to avoid the phosphate effect, the fatty acid was detected by ESI^-^-MS (Fig. 3B).

Among the reported AHL-acylases, only AiiC can deacylate the short chain C6-HSL [18]. In addition, PvdQand QuiP were verified to express C7-HSL-degrading activity. However, the substrate specificity of the Aac for AHLs is within the limits of more than six carbon-acyl chain (Table 4). Moreover, transferring the *aac *gene into *C. violaceum *CV026 significantly influenced violacein production and chitinase activity (Fig. 4). These results indicated that Aac has the potential to be a quorum- quenching agent.

Although the quorum-sensing signal for controlling the virulence factors of *R. solanacearum *is 3-OH-PAME, *solI *and *solR *are members of the 3-OH-PAME communication system regulon [25]. In our study, no 3-OH-PAME-degrading enzyme has been found using the BLASTP search when interrogated with the beta-hydroxypalmitate methyl ester hydrolase (BAF64544) [42]. There are SolI (NP 521405) and SolR (NP 521406) proteins of *R. solanacearum*GMI1000 sharing 86% and 87% identity, respectively, with that of SolI (O30920) and SolR (AAC45947) from *R. solanacearum*AW1. Because the SolI (O30920) synthesizes C6- and C8-HSLs, the GMI1000 strain might be expected to produce both of them. Although the physiological role of AHL-acylase in *R. solanacearum *is unclear yet, we consider that *R. solanacearum *might adopt a unique signal turnover system to control existing signals from a quorum-sensing mode [43]. The AHL-acylase would be a mechanism of interference to degrade exogenous signals produced by competitors. It may also be possible that these acylase prevent the accumulation of self generated signals, allowing the quorum response to switch off as is seen in *Agrobacterium tumefaciens *[43].

Recently, several reports indicated that quorum-quenching enzymes, such as lactonase, AHL-acylase, and oxidoreductase, have potential to be used as peptide drugs. Among them, AHL-lactonase has been applied in genetically engineered procedures to control plant diseases [35,44]. Eventually such enzymes would lead to the attenuation of the expression of quorum-sensing regulated functions in microorganisms. AHL-lactonases will alter the AHLs structure, thus mediating pH-dependent lactonolysis; however, lactonases-inactivated AHLs could be readily reverted to the active forms with lactone-ring closure [45]. This drawback would interfere with the development of AHL-lactonase as peptide drugs. Since AHL-acylases have none of the drawbacks described above, Aac could become a potential quorum-quenching agent in the near feature.

## Conclusion

This paper describes the identification of AHL-acylase, Aac, from *R. solanacearum*GMI1000 with ESI-MS mass spectrometry analysis and whole cell bioassay, together with the analysis of MIC test of aculeacin A. The results showed strong evidence that the Aac in *R. solanacearum*GMI1000 functions as an AHL-acylase and not an aculeacin A acylase. Thus, we consider that renaming the *aac *gene of *R. solanacearum*GMI1000 as "the *alaS *gene" is necessary in further studies for the purpose of clarity. Moreover, this is the first report to find an AHL-acylase in a phytopathogen.

## Abbreviations

(AHLs): *N*-acylhomoserine lactones; (C4-HSL): *N*-(butanoyl)-L-homoserine lactone; (C6-HSL): *N*-hexanoyl-L-homoserine; (C7-HSL): *N*-(heptanoyl)-L-homoserine lactone; (C8-HSL): *N*-(octanoyl)-L-homoserine lactone; (C10-HSL): *N*-(decanoyl)-L-homoserine lactone; (C12-HSL): *N*-(dodecanoyl)-L-homoserine lactone; (C14-HSL): N-tetradecanoyl-L-homoserine lactone; (3OC12-HSL): *N*-(3-oxo-dodecanoyl) -homoserine lactone; (HSL-OPA): Homoserine lactone-*o*-phthaldialdehyde; (HPLC): High-performance liquid chromatography.

## Authors' contributions

CNC conceived of the study, performed gene cloning and expression, MIC test, substrate specificities, statistical analysis, and drafted the manuscript. CJC performed the mass study and the data analyses. CTL prepared the crude proteins and performed the SDS-PAGE analysis. CYL initiated the ideas of the research, was involved in project design and coordination, and prepared the manuscript. All authors read and approved the final manuscript.
